# TAP2, a peptide antagonist of Toll-like receptor 4, attenuates pain and cartilage degradation in a monoiodoacetate-induced arthritis rat model

**DOI:** 10.1038/s41598-020-74544-5

**Published:** 2020-10-15

**Authors:** Hyewon Park, Jinpyo Hong, Yuhua Yin, Yongbum Joo, Youngmo Kim, Juhee Shin, Hyeok Hee Kwon, Nara Shin, Hyo Jung Shin, Jaewon Beom, Dong Woon Kim, Jinhyun Kim

**Affiliations:** 1 Department of Medical Science, Chungnam National University College of Medicine, Daejeon, Republic of Korea; 2 Department of Anatomy and Cell Biology, Brain Research Institute, Chungnam National University College of Medicine, Daejeon, 35015 Republic of Korea; 3 Department of Neuroscience and Physiology, Dental Research Institute, Seoul National University School of Dentistry, Seoul, Republic of Korea; 4 Department of Orthopedics, Chungnam National University College of Medicine, Daejeon, Republic of Korea; 5 Department of Rehabilitation Medicine, Seoul National University Bundang Hospital, Seongnam, Gyeonggi-do Republic of Korea; 6 Department of Internal Medicine, Chungnam National University College of Medicine, Daejeon, 35015 Republic of Korea

**Keywords:** Osteoarthritis, Osteoarthritis, Preclinical research, Medical research

## Abstract

Because inflammation in osteoarthritis (OA) is related to the Toll-like receptor 4 (TLR4) signaling cascades, TLR4 is a reasonable target for developing therapeutics for OA. Thus, we investigated whether TAP2, a peptide antagonist of TLR4, reduces the monoiodoacetate (MIA)-induced arthritic pain and cartilage degradation in rats. TLR4 expression of human OA chondrocytes and synoviocytes and the knee joint tissue of MIA-induced arthritis were evaluated. MIA-induced arthritic model using Sprague–Dawley rats (6 week-old-male) were treated with TAP2, a TLR4 antagonist, and evaluated with behavioral test, immunohistochemistry, and quantitative PCR. TLR4 was highly expressed in the knee joints of patients with OA and the MIA-induced rat model. Further, a single intraarticular injection of TAP2 (25 nmol/rat) molecules targeting TLR4 on day 7 after MIA injection dramatically attenuated pain behavior for about 3 weeks and reduced cartilage loss in the knee joints and microglial activation in the spinal dorsal horns. Likewise, the mRNA levels of TNFα and IL-1β, reactive oxygen species, and the expression of MMP13 in the knee joints of TAP2-treated rats was significantly decreased by TAP2 treatment compared with the control. Moreover, interestingly, the duration of OA pain relief by TAP2 was much longer than that of chemical TLR4 antagonists, such as C34 and M62812. In conclusion, TAP2 could effectively attenuate MIA-induced arthritis in rats by blocking TLR4 and its successive inflammatory cytokines and MMP13. Therefore, TAP2 could be a prospective therapeutic to treat patients with OA.

## Introduction

Osteoarthritis (OA) is a painful and disabling disease^1^. Although a few drugs, including acetaminophen, nonsteroidal anti-inflammatory drugs (NSAIDs), and intraarticular injections of corticosteroids are used to suppress OA pain^2,3^, unfortunately, no drugs are currently available with proven structure-modifying efficacy in OA. These NSAIDs sometimes are associated with serious adverse events, including an elevated risk of cardiovascular events, gastrointestinal bleeding, and renal toxicity^4^. The use of corticosteroids is also limited because it speeds up the progression of OA when injected repeatedly^5^, although a good effect is achieved in the short term following an intraarticular administration. Therefore, new therapeutics are required to overcome these limitations.

Toll-like receptor 4 (TLR4), a member of the toll-like receptor family that perceives pathogen-associated molecular patterns (PAMPs) expressed on infectious agents, recognizing lipopolysaccharide (LPS) in most gram-negative bacteria and subsequently inducing the activation of the innate immune system^6^. In OA, cartilage degeneration and loss, subchondral bone changes, and inflamed synovium are structural hallmarks. Altered chondrocytes produce inflammation-associated mediators, such as cytokines, chemokines, and proteolytic enzymes, which may further deteriorate the cartilage condition. Subchondral bone is also thickened, with the formation of bony outgrowths from the calcified cartilage layers and the bone surface. Moreover, the synovium becomes inflamed owing to the cartilage breakdown and secretes pro-inflammatory cytokines. When joint components are disrupted in OA, several types of endogenous molecules, such as fibronectin fragments^7^, hyaluronic acid^8^, S100A8, and S100A9^9^, are released into the joints cavities, bind to TLR4 on the cartilage and synovium of OA joints^10,11^, and then stimulate the secretion of cytokines, such as TNFα, IL-1β, and IL-6. Subsequently, these molecules propagate cartilage damage and further bind their receptors on the innervated sensory neurons projecting into the spinal cord and participate in the induction and modulation of pain.

Therefore, blocking TLR4 in the joints could effectively attenuate OA progression and diminish pain. Recently, a TLR4/MD2 specific peptide, TLR4 antagonistic peptide 2 (TAP2), was developed and shown to attenuate inflammatory responses to LPS^12^. Previously, we reported that an intrathecal administration of TAP2 decreased pain induced by sciatic nerve ligation and MIA injection into the knee joints through inhibiting the TLR4 signaling cascade in spinal microglia^13^. Because TLR4 is expressed in OA tissues and implicated in OA pathogenesis per se, we hypothesized that TAP2 could be effective in controlling pain and modifying the disease. Thus, we tested whether TLR4-targeted TAP2 could efficiently attenuate the pain and progression of cartilage degradation in an MIA-induced arthritic rat model, a common model for human OA.

## Materials and methods

### Animals

Sprague–Dawley rats (6 week-old-males, 150–200 g) were purchased from Daehan Bio Link (DBL, Chungcheongbuk-do, Korea) and acclimated for 1 week before the experiments in semi-specific pathogen free condition. All animals were housed three per cage in a controlled environment (23 ± 2 °C) with a 12-h light/dark cycle and food and water ad libitum. These experiments were approved by the Animal Care and Use Committee at Chungnam National University Hospital (CNUH-018-P0025) and followed the ethical guidelines of the National Institutes of Health and the International Association for the Study of Pain.

### Primary cultured human articular chondrocytes and synoviocytes

Knee joints tissues were obtained from 13 patients with OA during replacement surgery with the approval of the Institutional Review Board of Chungnam National University Hospital (2018-05-061). The written informed consents were obtained from the patients and all methods were performed in accordance with relevant guidelines and regulations. The tissues were washed with phosphate-buffered saline (PBS) to remove blood. The cartilage was then dissected from the knee bone and treated with hyaluronidase (500 μg/ml) for 10 min. Later, the tissues were chopped into smaller pieces using a scalpel and incubated with trypsin (8 mg/ml) for 2 h. Subsequently, the tissues were exposed to collagenase (1 mg/ml)/hyaluronidase (500 μg/ml) for an additional 2 h on a stirrer. Synovium were minced and and then incubate for 3 h in collagenase (1 mg/ml). Chondrocytes or synoviocytes obtained by the centrifugation at 12,000 rpm for 7 min were resuspended in complete culture media (DMEM with 10% fetal bovine serum (FBS) and 1% antibiotics) and seeded at 6–9 × 10^6^ cells/10 cm culture dish. The cultures were maintained for ~ 14 d by changing the medium every 3 d until the cells reached 80–90% confluency. Primary chondrocyte cultures at passage 0 and synoviocytes passaged three to four times were used for further experiments.

### MIA-induced arthritis model

An arthritis model was established in rats by the intraarticular injection of monoiodoacetate (MIA, Sigma, St. Louis, MO, USA) as described previously^14^. In brief, under isoflurane anesthesia (2%) in an oxygen mixture (Hana Pharm, Seoul, Korea), PBS (25 μl) or MIA (2 mg in 25 μl PBS) were delivered to the intraarticular space through the left patellar tendon using a 26.5G needle-installed Hamilton syringe. After 7 days, PBS or 25 nmol TAP2 (AMALDCFRWGWRMWCSSG, Peptron, Daejeon, Korea) was then administrated to rats randomly with the same injection method to observe their analgesic effects on OA pain. To compare an analgesic effect of TAP2 with other TLR4 antagonists, C34 (5373, Tocris Bioscience, MO, USA, Ellisville) or M62812 (5705, Tocris Bioscience) was administered intraarticularly at the same concentration as TAP2. Afterward, the animals were evaluated by behavioral tests, histology, and molecular analysis on the indicated days. There were no adverse events to animals during the experiments. Further, the distribution and absorption of TAP2 in the knee joint of MIA-induced rats was investigated using fluorescein isothiocyanate (FITC)-conjugated TAP2 (FITC-TAP2, Peptron).

### Pain behavior test with von Frey filaments

Pain behavior was evaluated with von Frey filaments as described previously^15^. In short, mechanical allodynia was detected with von Frey filaments (NC12775-99, Touch Test Sensory Evaluators, 20-Piece Hand Kit, North Coast Medical, Morgan Hill, CA, USA) one day (baseline) prior to injection, and days 3, 5, 7, 8, 9, 10, 12, 14, 17, 21, 24, and 28 following PBS- or MIA-administration^16^. The withdrawal threshold of each paw was measured with von Frey filaments applied accurately on the plantar surface of the hind paws three times for a duration of 1–2 s by the up-and-down testing paradigm^17^. Filament (10-g) was used for setting the baseline and a 4-g filament was applied firstly after surgery to judge the development of neuropathic pain. If a positive response occurred, the next smaller filament was used; if a negative response was observed, the next higher one was applied^16^.

### Tissue preparation, histology and immunohistochemistry

The rats were anesthetized with sodium pentobarbital (100 mg/kg, i.p.), perfused with PBS (pH 7.4), followed by perfusion with ice-cold 4% paraformaldehyde (Merck, Darmstadt, Germany) in PBS for 10 min using a peristaltic pump at a rate of 20 ml/min. The knee joints and spinal cord fragments (L4–5) were immediately dissected and bathed in the same fixative overnight at 4 °C. The next day, the knee joints were dissected to remove muscle and transferred to a decalcifying buffer containing 7% AlCl_3_, 5% formic acid and 8.5% HCl for 2 d at 4 °C. Then, the knee joint and spinal cord tissue were treated by immersion in a sucrose gradient from 10 to 30%^18^. Later, OCT-embedded tissues were sectioned sagittally at a thickness of 35-µm using a cryostat (CM1950, Leica Biosystems, Wetzlar, Germany), mounted on gelatin coated-slides, stored at − 70 °C^14,19^, and used for immunohistochemistry. The spinal cords were preserved at 4 ℃ in a storage buffer (30% glycerol and 30% ethylene glycol in PBS).

For cartilage Safranin O staining of the knee joints, the sections were stained in 0.1% Fast green solution for 5 min, rinsed quickly in 1% glacial acetic acid for 10 s, and stained further in 0.1% Safranin O solution for 5 min. Subsequently, the tissues were dehydrated with a series of alcohols, cleared with xylene and mounted with Permount solution (Thermo Fisher Scientific, Waltham, MA, USA). Images were taken under a bright microscope (ECLIPSE E600 POL, Nikon, Tokyo, Japan) and analyzed by Image J (National Institutes of Health, Bethesda, MD, USA). Knee joint histology was also evaluated using OARSI score system^20^.

For fluorescent immunostaining, the sections on the slides were first blocked with a blocking buffer (5% normal serum and 0.3% Triton X-100 in PBS) for 1 h at room temperature. Thereafter, the sections were incubated with a mixture of primary antibodies (anti-TLR4, mouse, 1:500, Cat. No. ab22048, Abcam, Cambridge, United Kingdom; anti-collagen-type II, rabbit, 1:400, #ab116242, #ab34712, Abcam; anti-Thy1, rabbit, 1:400, #13801S, Cell Signaling Technology, Danvers, MA, USA) diluted in blocking buffer and followed by the respective secondary antibodies conjugated with either FITC or Cy3 (1:400, Jackson ImmunoResearch, West Grove, PA, USA) diluted in the same blocking buffer. Negative controls without primary antibodies were also stained. Finally, the tissues were covered with mounting medium (Biomeda, Foster City, CA, USA). Images were acquired under a laser scanning microscope (TCS SP8, Leica Microsystems, Wetzlar, Germany) and used for additional data analysis.

For immunohistochemistry, the tissues were initially exposed to 0.3% H_2_O_2_ in PBS for 10 min to remove endogenous peroxidase and then incubated with the same blocking solution used in the fluorescent immunostaining. Later, primary antibodies were applied to the knee joint (anti-MMP13, mouse, 1:100, #MA5-14238, Thermo Fisher Scientific) and spinal cord (anti-Iba-1, rabbit, 1:400, # 019-19741, Wako) sections overnight at 4 °C, followed by incubation with the corresponding biotinylated secondary antibodies and streptavidin peroxidase complex (Vector Laboratories, Inc., Burlingame, CA, USA). Lastly, the specimens were visualized in a solution of diaminobenzidine (DAB, Sigma) and hydrogen peroxide and dehydrated and mounted on slide glasses using the Permount solution. Images were captured under a bright microscope (ECLIPSE E600 POL, Nikon, Tokyo, Japan) and used for further analysis. The immunodensities in the graphs were quantified by the Image J program software.

### ROS staining of rat cartilage

The cartilage tissues were labeled with dihydroethidium (DHE) at a final concentration of 10 μM and incubated for 10 min. The tissues were carefully washed with cold PBS three times and imaging was conducted using a blue and green filter in a fluorescence microscope (Leica DMi8, Leica Microsystems). An H_2_O_2_-specific sensor was applied for the detection of intracellular H_2_O_2_ according to the manufacturer's protocol (Sigma-Aldrich, #MAK164). An H_2_O_2_ standard was run and measured similarly using the H_2_O_2_ sensor. The immunodensities in the graphs were quantified by the Image J program software.

### Quantitative PCR and RT-PCR

Human chondrocytes and synovial cells were treated with TAP2, C34 and M62812 at concentrations of 10 μM and 100 μM for 3 h, respectively. After that, LPS (0.1 ng/ml or 100 ng/ml) was treated for 3 h. In addition, cartilage was scraped from rat joint 10 days after injection of TAP2 into MIA-induced rat knee. Total RNA was isolated from human chondrocytes, human synoviocytes, and rat cartilage using TRIzol Reagent according to the manufacturer’s protocol (Gene All, RoboExTM). The RNA concentration was quantified using Nanodrop spectrometer (Thermo Scientific). cDNA synthesis was then conducted in a 20 µl reaction using the TOPscript RT DryMix (Enzynomics, Daejeon, Korea).

Quantitative real-time PCR (qPCR) was performed in 10 μl reactions volumes (10 pM primer, 4 μl cDNA, and 5 μl SYBR Green 2X mixture) and the following condition^21^: 95 °C for 10 min, then 40 cycles of 95 °C for 15 s and 60 °C for 1 min using the AriaMx Realtime PCR System (Agilent Technologies, Santa Clara, CA, USA). The human primer sequences (Cosmogenetech, Daejeon, Korea) used for the QPCR were as follows: GAPDH, forward: 5′-CAA TGA CCC CTT CAT TGA CC-3′, reverse: 5′-TTG ATT TTG GAG GGA TCT CG-3′; TNFα, forward: 5′-TCA ACC TCC TCT CTG CCA TC-3′, reverse: 5′-CCA AAG TAG ACC TGC CCA GA-3′; IL-1β, forward: 5′-CTG TCC TGC GTG TTG AAA GA-3′, reverse: 5′-TTC TGC TTG AGA GGT GCT GA-3′; IL-6, forward: 5′-AGG AGA CTT GAA TGG TGA AA-3′, reverse: 5′-CAG GGG TGG TTA TTG CAT CA-3′; MMP3, forward: 5′-CTG GAC TCC GAC ACT CTG GA-3′, reverse: 5′-CAG GAA AGG TTC TGA AGT GAC C-3′; MMP13, forward: 5′-AAC ATC CAA AAA CGC CAG AC-3′, reverse: 5′-GGA AGT TCT GGC CAA AAT GA-3′, TLR1, forward: 5′-AGA TTT CTT GCC ACC CTA CTG-3′, reverse: 5′-GCT CAA CCC CAG AAA TTT TAG-3′, TLR2, forward: 5′-AAC TTA CTG GGA AAT CCT TAC-3′, reverse: 5′-AAA AAT CTC CAG CAG TAA AAT-3′, TLR4, forward: 5′-TCA CCT GAT GCT TCT TGC TG-3′, reverse: 5′-CAG GGC TTT TCT GAG TCG TC-3′, TLR5, forward: 5′-AGC TTC AAC TAT ATC AGG ACA-3′, reverse: 5′-TGG TTG GAG GAA AAA TCT AT-3′, TLR9, forward: 5′-CGC CCT GCA CCC GCT GTC TCT-3′, reverse: 5′-CGG GGT GCT GCC ATG GAG AAG-3′. The rat primer sequences (Cosmogenetech) used for the QPCR were as follows: GAPDH, forward: 5′-CAA TGA CCC CTT CAT TGA CC-3′, reverse: 5′-TTG ATT TTG GAG GGA TCT CG-3′; TNFα, forward: 5′-TCA ACC TCC TCT CTG CCA TC-3′, reverse: 5′-CCA AAG TAG ACC TGC CCA GA-3′; IL-1β, forward: 5′-CTG TCC TGC GTG TTG AAA GA-3′, reverse: 5′-TTC TGC TTG AGA GGT GCT GA-3′; IL-6, forward: 5′-AGG AGA CTT GAA TGG TGA AA-3′, reverse: 5′-CAG GGG TGG TTA TTG CAT CA-3′, TLR1, forward: 5′-GTG TGC AGC TGA TTG CTC AT-3′, reverse: 5′-CTG GCA CCT CCA GAA GAA AG-3′, TLR2, forward: 5′-CCC TTG TCC TGT TGA TCG TT-3′, reverse: 5′-CAT GAG GTT CTC CAC CCA GT-3′, TLR4, forward: 5′-GCT TCA ATG GTG CCA TTT TT-3′, reverse: 5′-TTG CCA GCC ATT TTT AAG GT-3′, TLR5, forward: 5′-GCT CGC TTA GAC CTG TCT GC-3′, reverse: 5′-AGA GAG TGT TTT GCC CTC CA-3′, TLR9, forward: 5′-GGA CGA GGA GGA CCT TTA CC-3′, reverse: 5′-AGG CCA GAC TGC TCC AGT TA-3′. The mRNA level of each target gene was normalized to that of GAPDH mRNA. The fold change in the mRNA level was calculated by the 2^−ΔΔ*C*t^ method as described previously^22^.

In addition, RT-PCR was carried out in 20 μl reactions (10 μl master 2X mixture, 4 μl cDNA, and 5 pM primer) with the general conditions (1 cycle of 95 °C for 5 min; 25 cycles of 95 °C for 30 s, 55 °C for 30 s, 72 °C for 30 s; and 1 cycle of 72 °C for 5 min) using a PCR cycler (Thermo Fisher Scientific). The human primer sequences (Cosmogenetech) used for the PCR were as follows: TLR4, forward: 5′-TCA CCT GAT GCT TCT TGC TG-3′, reverse: 5′-CAG GGC TTT TCT GAG TCG TC-3′. The PCR products were separated by electrophoresis on 1.5% agarose gel and visualized with a NaBI gel documentation system (NEO Science, Kyunggi-do, Korea).

### Statistical analysis

The data are expressed as the mean ± standard error of the mean (SEM). The statistical significance of the differences between two groups or multiple groups was compared by unpaired Student’s *t* test or one-way analysis of variance (ANOVA) followed by an appropriate multiple comparison test. *P* values of < 0.05 were considered statistically significant. All statistical analyses were performed using Prism 6 (GraphPad Software, La Jolla, CA, USA).

## Results

### TLR4 is highly expressed in knee joints of patients with OA

**Figure 1:**
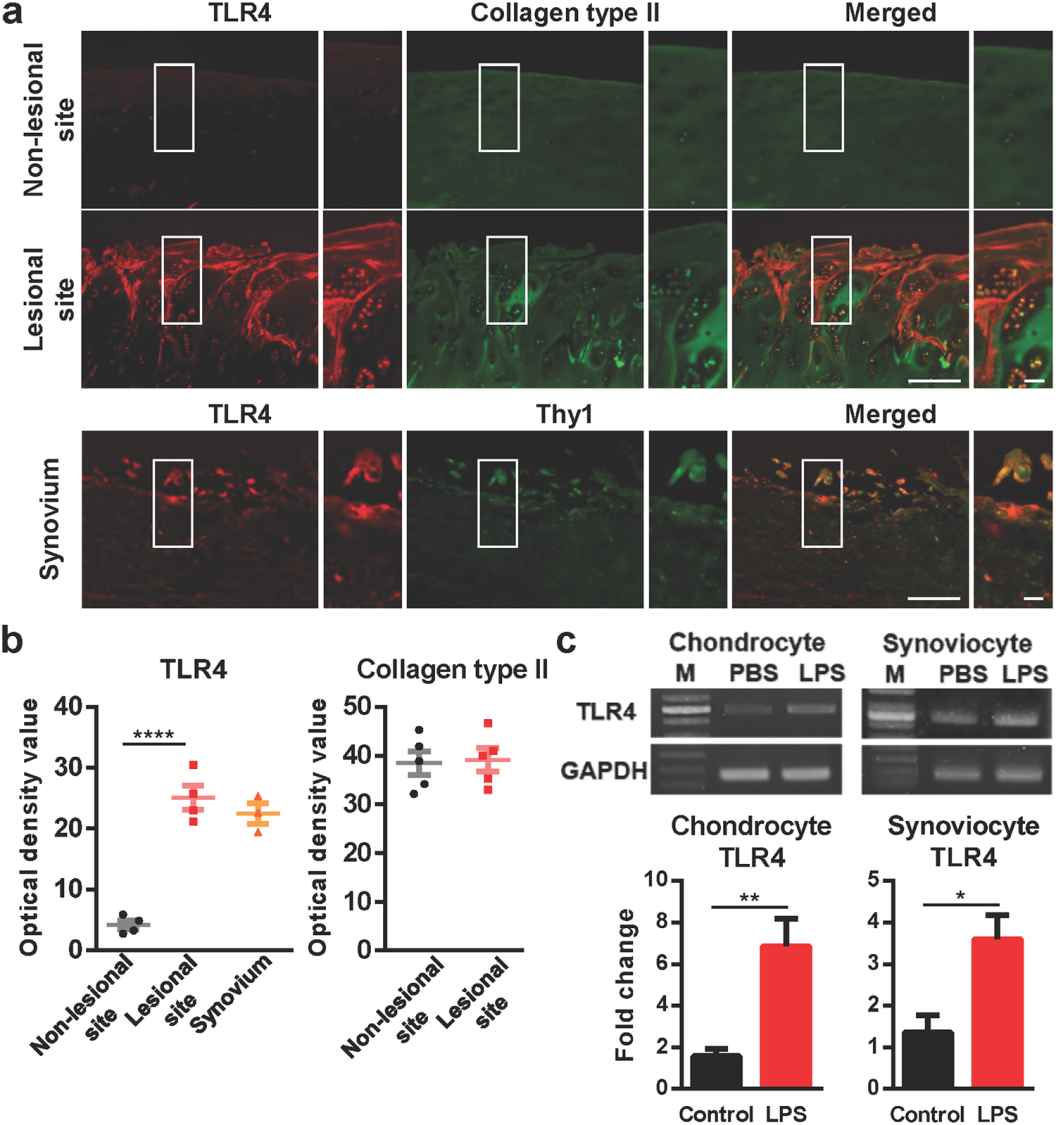
TLR4 is mainly expressed in the chondrocytes and synoviocytes of the knee joints of patients with OA. (**a**) Paraffin sections of the knee joints from patients with OA were co-immunostained with anti-TLR4 antibodies and anti-COL2A1 antibodies (chondrocyte marker) or anti-THY1 antibodies (synoviocyte marker). Scale bar = 100 μm (left), 20 μm (right) (**b**) The intensity of TLR4 and Collagen type II staining was quantified in cartilage and synovium. The data are expressed as the mean ± SEM (one-way ANOVA test, ****P < 0.0001 vs. Non-lesional site). (**c**) Primary cultured human chondrocytes and synoviocytes were stimulated by LPS (10 ng/ml, 3 h). TLR4 induction was determined at the mRNA level by RT-PCR and quantitative PCR. GAPDH was used as a control. The data are expressed as the mean ± SEM (unpaired t test, **P < 0.01, *P < 0.05 vs. Non-lesional site).

First, to investigate whether TLR4 expression was changed in patients with OA, we conducted immunohistochemical analysis with anti-TLR4 antibodies on knee joints from patients with OA. TLR4 was highly expressed in the superficial chondrocytes clustering in damaged OA cartilage and synoviocytes of OA synovium (Fig. 1a,b, Supplementary Fig. S1). Moreover, we confirmed the expression of TLR4 in primary cultured chondrocytes and synoviocytes from OA tissue by RT-PCR and qPCR (Fig. 1c, Supplementary Fig. S1b), although other TLRs were also expressed (Supplementary Fig. S1c). The TLR4 expression in both chondrocytes and synoviocytes was increased by LPS stimulation (Fig. 1c, Supplementary Fig. S1b), Together, these data suggest that TLR4 was highly expressed in the knee joints of patients with OA.

### MIA injection upregulates TLR4 expression in knee joints, pain behavior and microglial activity

**Figure 2:**
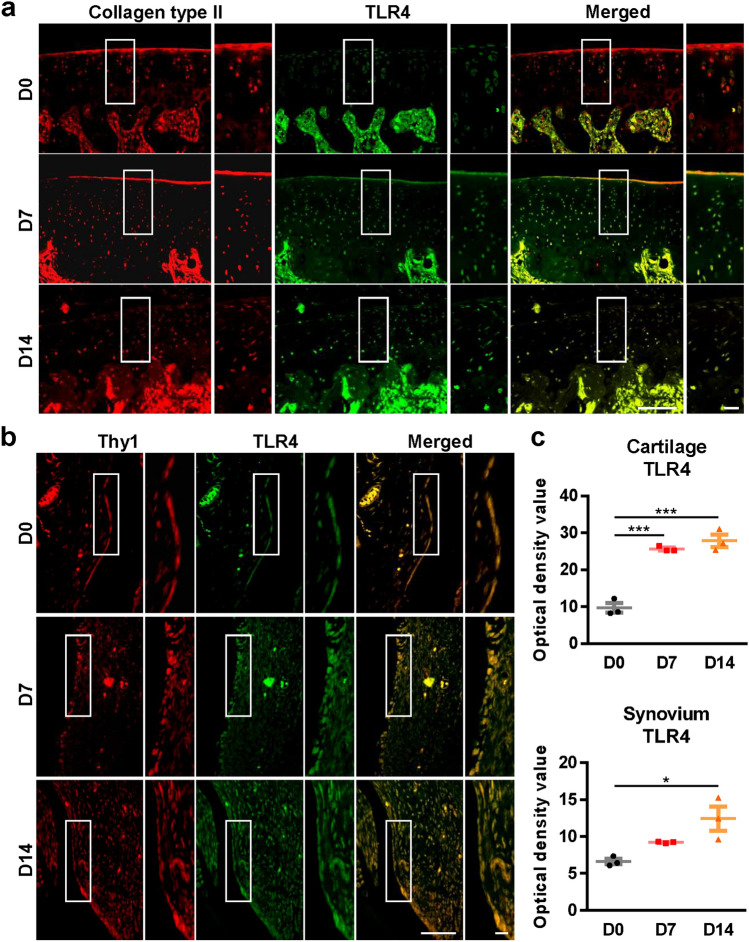
TLR4 is highly upregulated in the chondrocytes and synoviocytes of knee joints in MIA-induced rats. Knee joint tissues from MIA-induced rats (n = 3) were co-immunostained with (**a**) anti-TLR4 antibodies and anti-collagen type II antibodies (chondrocyte marker) or (**b**) anti-TLR4 antibodies and anti-Thy1 antibodies (synoviocyte marker). Scale bar = 100 μm. (**c**) The intensity of TLR4 staining was quantified in cartilage and synovium. The data are expressed as the mean ± SEM (one-way ANOVA test, ***P < 0.001, *P < 0.05 vs. D0).

To establish a rat model of OA, MIA was delivered into the knee of the hind legs, which has been shown to induce cartilage damage by the gradual death of chondrocytes in the knee joint. When MIA (2 mg in 25 μl PBS) was injected into the knee joints, the articular cartilage in the knee joints was progressively lost from day 7 after MIA-injection observed by Safranin O and Fast green staining (Supplementary Fig. S2a). This cartilage degradation subsequently led to mechanical allodynia and microglial activation of the ipsilateral horn of the spinal cord (Supplementary Fig. S2b, c, d). In MIA-administered rats, TLR4 expression increased in cartilage and synovium on days 7 and 14 after MIA-injection compared with chondrocytes on day 0 (Fig. 2a–c, Supplementary Fig. S3). Because the increase in TLR4 expression in the chondrocytes and synoviocytes of rats following MIA-administration was similar to that in human OA, we used the animal model to validate the effect of TLR4 antagonists for OA.

### TAP2 is localized in the chondrocytes and synoviocytes of knee joints in MIA-induced arthritic rats

**Figure 3:**
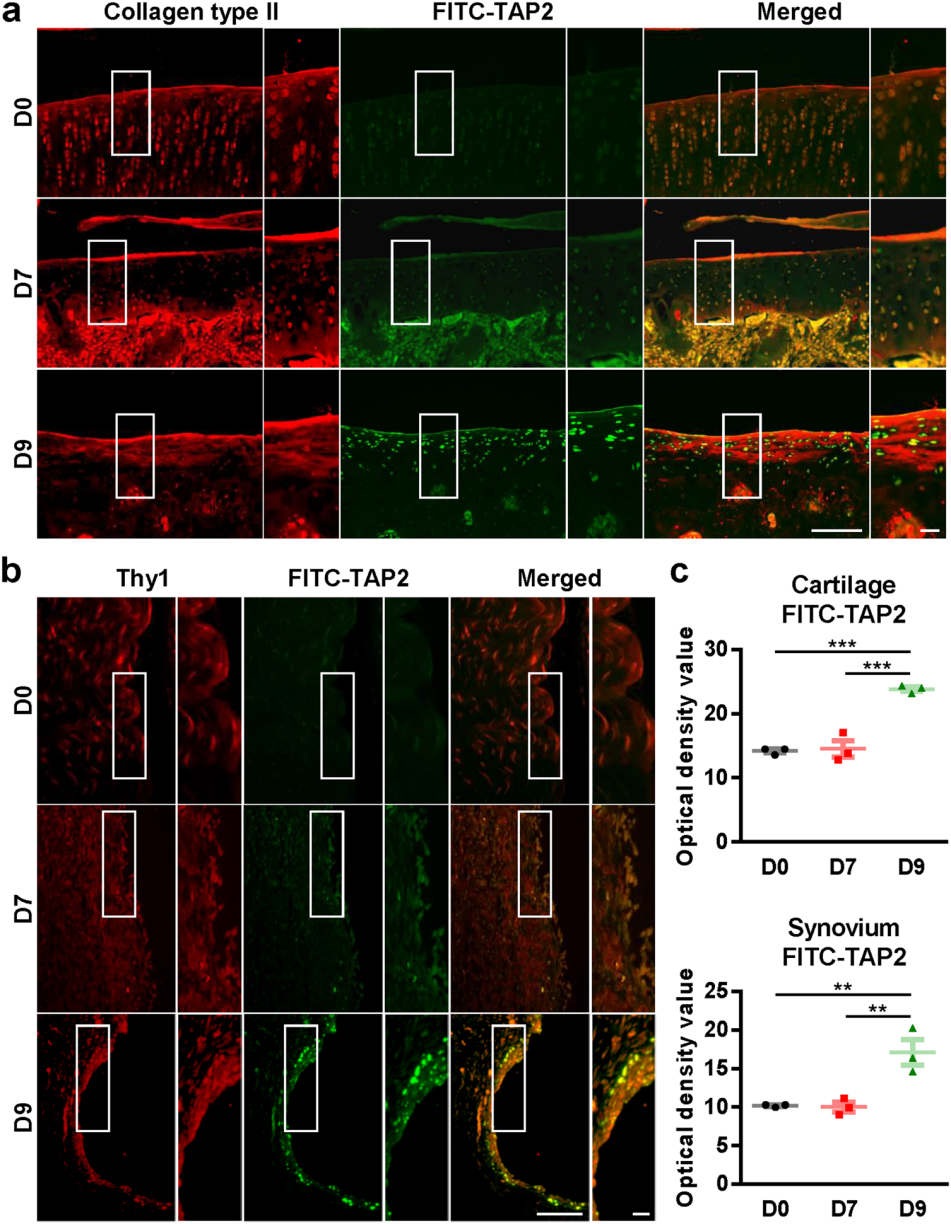
TAP2 is localized in the chondrocytes and synoviocytes of arthritic joints of rats. FITC-conjugated TAP2 was delivered to the knee joints in rats (n = 3) on day 7 post-MIA injection. After 48 h, the knee joints were immunostained with (**a**) anti-collagen type II (chondrocytes) or (**b**) anti-Thy1 (synoviocytes). Scale bar = 100 μm (left). (**c**) The intensity of FITC-TAP2 staining was quantified in cartilages and synovium. The data are expressed as the mean ± SEM (one-way ANOVA test, ***P < 0.001, **P < 0.01 vs. D9).

Prior to the application of TAP2 to MIA-induced OA rats, to trace the distribution and uptake of TAP2 in the knee joints of the rats, FITC-conjugated TAP2 was delivered into the knee joints on day 7 after MIA injection. We observed that FITC green fluorescence FITC was mainly localized in the cartilage and synovium of the knee joints two days post-FITC-TAP2 injection, which demonstrated that TAP2 could target both the chondrocytes and synoviocytes of arthritic joints (Fig. 3a–c).

### Intraarticular injection of TAP2 attenuates pain behavior, cartilage loss, and microglial activity in the spinal cords of MIA-induced arthritic rats

**Figure 4:**
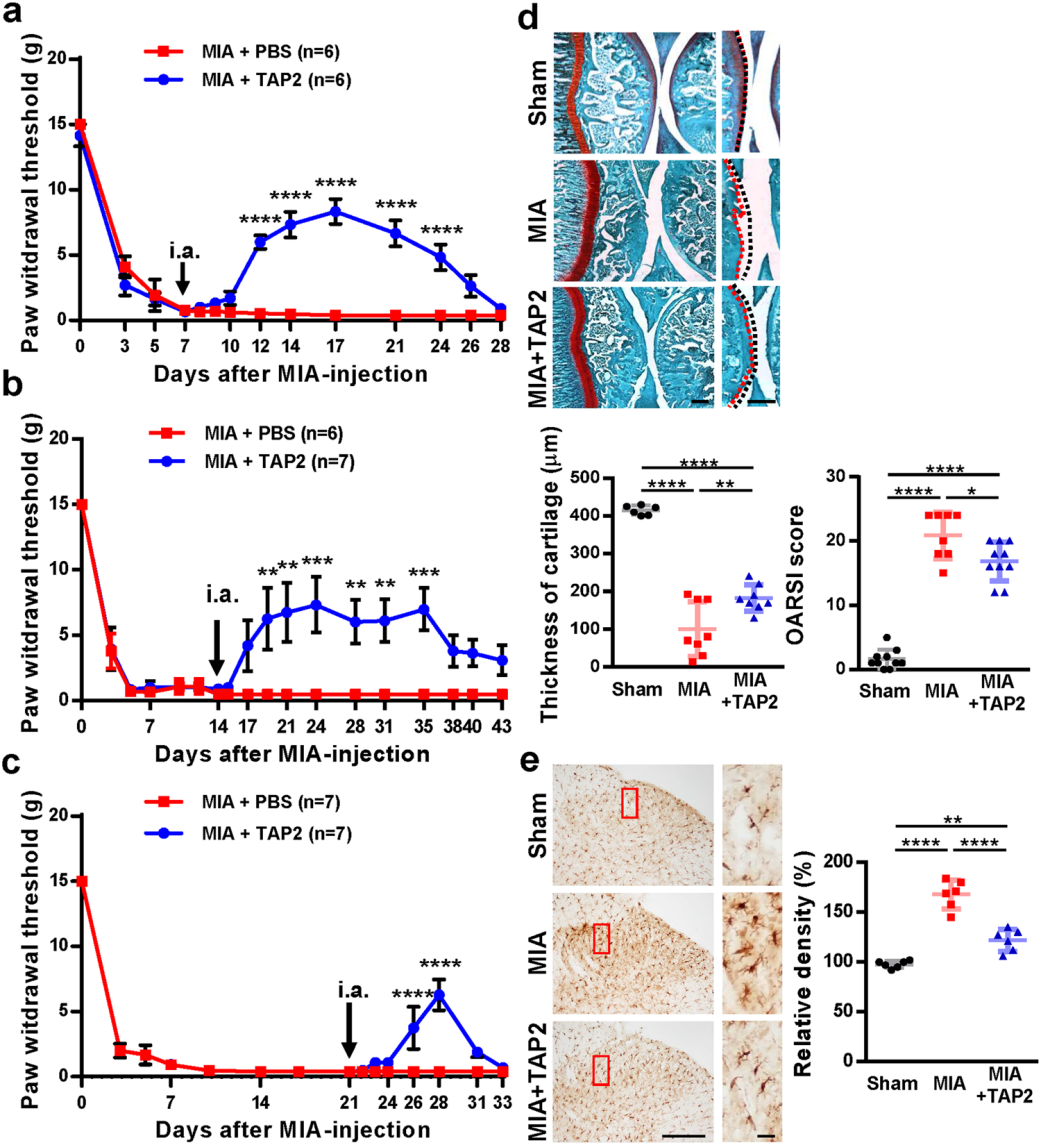
Intraarticular injection of TAP2 attenuates pain, cartilage loss and microglial activity. (**a**) PBS or TAP2 (25 nmol/rat) was delivered to the intraarticular space of MIA-induced rats on day 7 after MIA injection. Then, animals were evaluated by von Frey filaments to measure the withdrawal threshold for 3 weeks. (**b**, **c**) To test the analgesic effect of TAP2 on chronic OA pain, TAP2 (25 nmol/rat) was administrated once intraarticularly to MIA-induced rats on day 14 after MIA injection (**b**) or on day 21 (**c**) and evaluated in the same manner. The data are expressed as the mean ± SEM (two-way ANOVA test, ****P < 0.0001, ***P < 0.001, **P < 0.01, *P < 0.05 vs. MIA + PBS). (**d**) Knee joints were prepared on day 10 after PBS or TAP2 injection, sectioned, and stained in Fast green and Safranin O solution (n = 3). The cartilage thickness in the knee joints was then measured using ImageJ software. Black dashed line (undamaged area), red dashed line (damaged area). OARSI score also measured in the arthritic joints of rat. The data are expressed as the mean ± SEM (one-way ANOVA test, ****P < 0.0001, **P < 0.01, *P < 0.05 vs. MIA). Scale bar = 1 mm (left), 100 μm (right). (**e**) Sections (L4–5) of the spinal cords from the same animals (Fig. 4d, n = 3) were immunostained with anti-Iba1 antibodies. Scale bar = 100 μm (left), 50 μm (right). The data are expressed as the mean ± SEM (one-way ANOVA test, ****P < 0.0001 vs. MIA).

Next, to determine whether TAP2 could mitigate arthritic pain in rats, MIA-induced rats were randomly divided into two groups and treated by intraarticular injections of PBS (20 μl) or TAP2 (25 nmol in 20 μl PBS) on day 7 after MIA injection. According to the von Frey filaments test, the pain withdrawal threshold was increased after TAP2 injection, but not after PBS injection. The results indicated that the analgesic effect of TAP2 was maintained for approximately 4 weeks compared with the control group (Fig. 4a). Treatment with TAP2 late in the disease course, on day 14 (Fig. 4b) or 21 (Fig. 4c), also showed analgesic effects.

In the evaluation of knee joint tissues of MIA-induced rats treated on day 7 and euthanized on day 28, cartilage thinning and OARSI score was attenuated in the knee joints treated with TAP2 compared with those treated with PBS (Fig. 4d). Microglial activation in spinal cord was also decreased (Fig. 4e). Reduced cartilage degradation and microglial activation may contribute to analgesia.

### TAP2 efficiently suppresses the expression of proinflammatory cytokines in human chondrocytes and synoviocytes and rat arthritic joints

**Figure 5:**
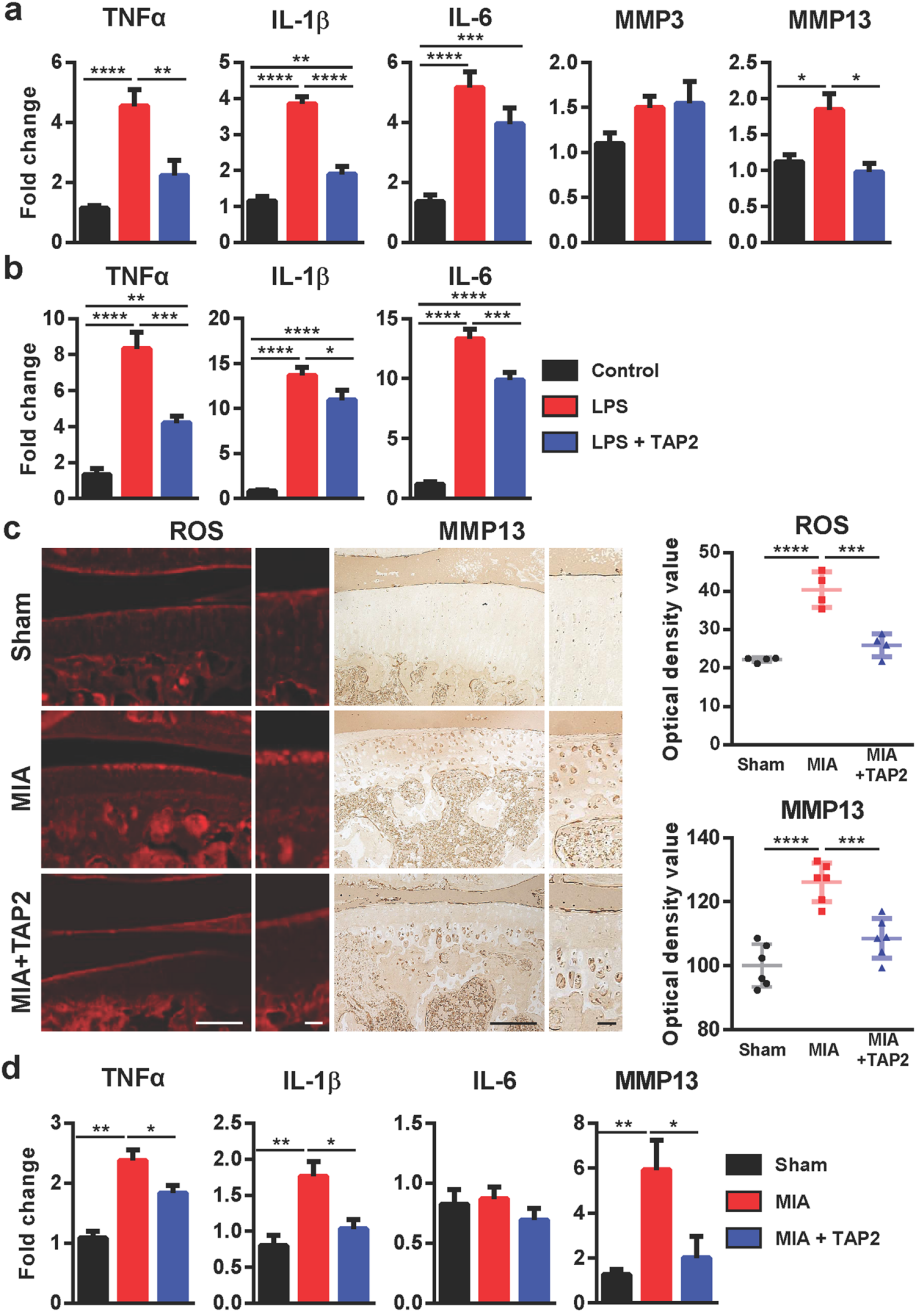
TAP2 efficiently suppresses the expression of proinflammatory cytokines and MMP13. (**a**) Primary human chondrocytes were incubated with TAP2 (10 μM, 3 h) prior to LPS treatment (0.1 ng/ml, 3 h) or (**b**) primary human synoviocytes were incubated with TAP2 (100 μM, 3 h) prior to LPS treatment (100 ng/ml, 3 h). Then, total RNA was isolated from the cells and utilized for the analysis of the inflammatory mediators, such as TNF-α, IL-1β, IL-6, MMP3, or MMP13, in chondrocytes and TNF-α, IL-1β, and IL-6 in synoviocytes by quantitative PCR. The data are expressed as the mean ± SEM (one-way ANOVA test, ****P < 0.0001, ***P < 0.001, **P < 0.01, *P < 0.05 vs. LPS + TAP2). (**c**) On day 3 post-injection of PBS and TAP2, the knee joint tissues of MIA-induced arthritic rats (n = 3) were incubated with DHE solution (1 μM) to visualize ROS or immunostained with an anti-MMP13 antibody. The intensity of ROS attaining or MMP13 was quantified in cartilages using ImageJ. The data are expressed as the mean ± SEM (one-way ANOVA test, ****P < 0.0001, ***P < 0.001 vs. MIA). (**d**) Total RNA from the knee joint cartilage of MIA-induced arthritic rats, MIA arthritis rats treated with TAP2, and control animals was isolated and analyzed by quantitative PCR (n = 3 per each group). The data are expressed as the mean ± SEM (one-way ANOVA test, **P < 0.01, *P < 0.05 vs. MIA).

To understand the mechanism underlying the reduction in pain and cartilage degradation by TAP2, we examined the expression levels of proinflammatory mediators, such as TNFα, IL-1β, IL-6, matrix metalloproteinase (MMP) 3, and MMP13 in LPS-treated human chondrocytes. LPS treatment increased the mRNA levels of TNFα, IL-1β, IL-6, and MMP13 in human chondrocytes, which was significantly inhibited by TAP2 treatment except IL-6. The expression of IL-6 tended to decrease although it was not significant (Fig. 5a). MMP3 expression in chondrocytes was not changed by either LPS treatment or TAP2 treatment. LPS treatment also induced the mRNA levels of TNFα, IL-1β, and IL-6 in human synoviocytes, which were diminished by TAP2 treatment (Fig. 5b). Our findings suggest that TAP2 could inhibit the gene expression of proinflammatory cytokines in human chondrocytes and synoviocytes, as well as MMP13, in human chondrocytes. Furthermore, TAP2 also suppressed LPS-induced TLR4 expression in human chondrocyte and syniviocyte (Supplementary Fig. S4a, b).

In the arthritic joints of rats, ROS production and MMP13 expression by MIA injection were decreased by TAP2 treatment (Fig. 5c). The expression levels of TNFα, IL-1β, and MMP13 in arthritic cartilages of rats were diminished by TAP2 treatment (Fig. 5d). Moreover, the expression of TLR4 induced by MIA injection was decreased by TAP2 treatment (Supplementary Fig. S4c, d).

### Comparison with other TLR antagonists

**Figure 6:**
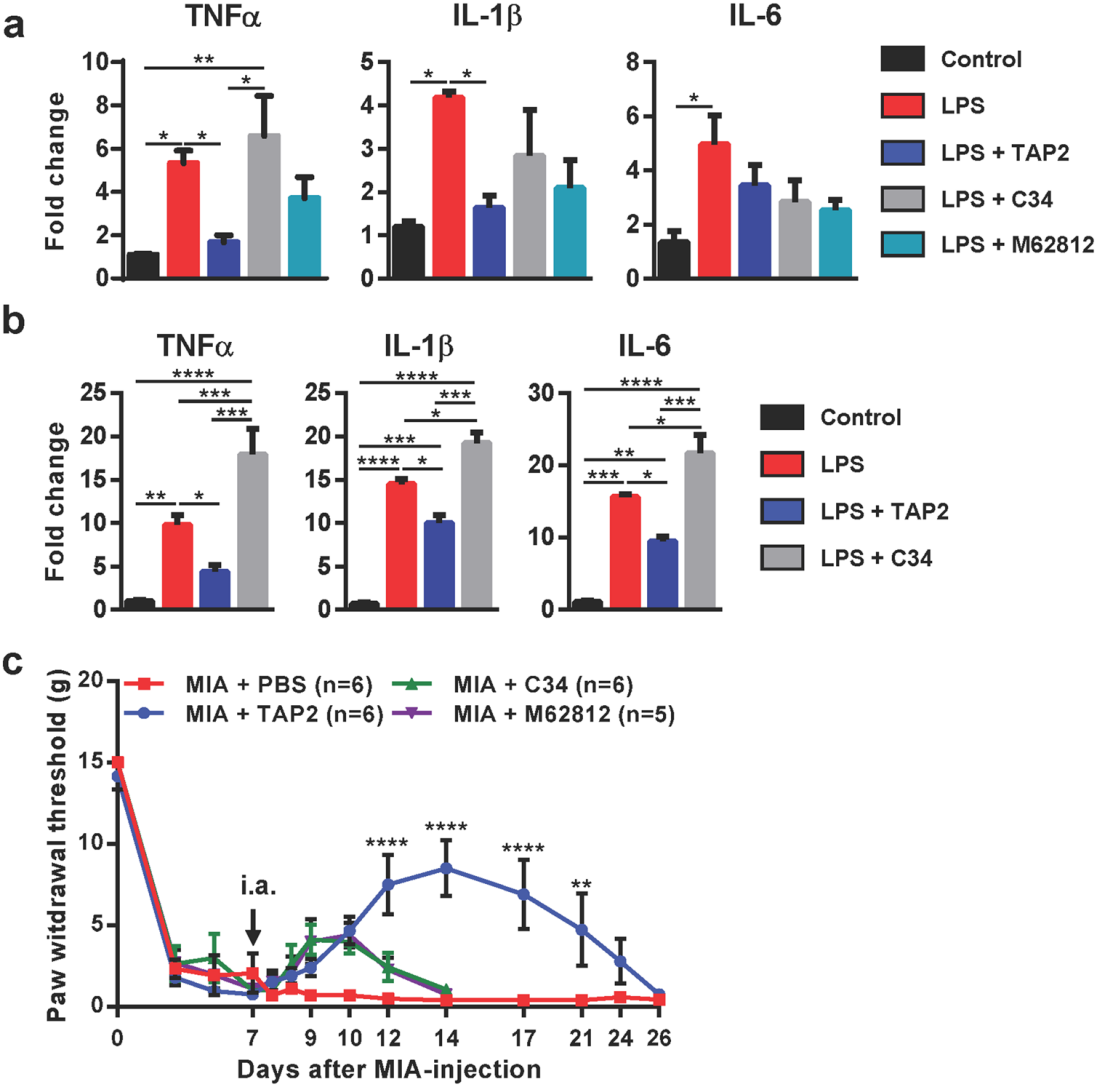
TAP2 shows better efficacy than other TLR4 antagonists, C34 or M62812. (**a**) Primary human chondrocytes of OA joints were incubated with TAP2 (10 μM), C34 (10 μM) or M62812 (10 μM) for 3 h prior to LPS treatment (0.1 ng/ml, 3 h) or (**b**) human synoviocytes of OA joints was incubated with TAP2 (100 μM) or C34 (100 μM) for 3 h prior to LPS treatment (100 ng/ml, 3 h). Then, total RNA was isolated from the cells and utilized for the analysis of the inflammatory mediators, such as TNF-α, IL-1β, or IL-6 in chondrocytes and synoviocytes by quantitative PCR. The data are expressed as the mean ± SEM (one-way ANOVA test, ***P < 0.001 vs. LPS + TAP2). (**c**) To compare the efficacy of TAP2 with C34 or M62812, each substance was injected directly into the knee joint space of MIA-induced animals 7 days after MIA injection. Then, the animals were evaluated by von Frey filaments to measure the withdrawal threshold for 3 weeks. The data are expressed as the mean ± SEM (two-way ANOVA test, ****P < 0.0001, **P < 0.01 vs. MIA).

We compared the effects of TAP2 and other TLR4 antagonists, C34 and M62812 on chondrocytes and C34 on synoviocytes at the same concentration. TAP2 suppressed LPS-induced production of TNFα and IL-1β in chondrocyte whereas C34 or M62812 did not. Moreover, TAP2 also decreased LPS-induced production of TNFα, IL-1β and IL-6 in synovium whereas C34 did not (Fig. 6a,b). In addition, we treated MIA-induced arthritis model with C34 and M62821, to compare to the analgesic effect of TAP2. Interestingly, the duration of analgesia with C34 and M62812 lasted about 2 weeks whereas the analgesic effect of TAP2 lasted 4 weeks. The prolonged analgesic effects of TAP2 compared with other TLR4 inhibitors may be clinically meaningful for the treatment of arthritic pain (Fig. 6c).

## Discussion

In this study, we tested whether an antagonistic peptide of TLR4 could be an effective therapeutic for the pain and cartilage degradation of arthritis because TLR4-mediated inflammation contributes to the induction of cartilage damage and propagation of arthritic pain. An intraarticular administration of TAP2 into MIA-induced arthritic rat model on day 7 or 14 after surgery markedly reduced the pain and cartilage degradation, with the loss of microglial activation and downregulation of ROS, MMP13, and IL-1β in the joints. Likewise, TAP2 also decreased the production of inflammatory cytokines or MMP in primary cultured human chondrocytes and synoviocytes.

OA is strongly associated with chronic and low-grade inflammation. Clinical observations showed that many patients with knee OA had joint effusion. A considerable number of patients with knee OA had radiographic changes of effusion and synovitis in MRI associated with, not only with their clinical symptoms, such as pain and stiffness, but also with cartilage loss after several years^23^. Low-grade inflammation probably comes from various sources, such as infiltrating of macrophages, mast cells, and T lymphocytes in the synovium and adipose tissue, and cytokines and TLR ligands in synovial fluid^24^. Thus, many researchers believe that inflammation is one of the key steps in cartilage damage and OA progression. Furthermore, many studies have demonstrated that TLR4-mediated inflammation in chondrocytes, synoviocytes, bone, and fat pads is the important elements in OA^25^. Several studies have explored TLRs in macrophages in OA synovium^26,27^. TLRs expression in lesioned articular cartilage was associated with cartilage degradation grade and TLR4 stimulation suppressed biosynthesis in chondrocytes^28,29^. Chronic pain in patients with OA primarily depends on the activation of sensory neurons innervating the affected joint^30^. Some evidence has shown that TLR4 on small-diameter, somatosensory neurons is directly involved in pain conduction and regulation^31^. In addition, pain in arthritic animal models was shown to be closely related to TLR4 signaling pathways^32^. This evidence suggests that TLR4 is a feasible target for OA management.

TLR4 and its accessory molecules (MD2, CD14, or CD36), intracellular components of TLR-mediated signaling pathways (MyD88, NF-κB), and other downstream inflammatory mediators can be used as targets for intervention. Several strategies have been suggested to control TLR4 signaling in diseases. Although new chemicals inhibiting the interaction of TLR4 and accessory molecules MD2, such as eritoran, C34 and C35, has been developed, these TLR4 antagonists were tested only in sepsis^33,34^. Drug intervention with agents such as EGb761 or resveratrol has been used in animal models to target TLR4 and its related signaling pathway in the treatment of OA^35,36^. However, direct targeting of TLR4 has not yet been properly exploited as a therapeutic approach to treat patients with OA.

There is some controversy about the role of TLR4 in OA pathogenesis in animal models. TLR4 knock-out mice did not show any difference in OA progression compared with wild-type mice in a traumatic OA model^37^. However, many animal studies showed the upregulation of TLR4 in arthritic joints. TLR4 expression was associated with disease course^38^. TLR4 is known to promote obesity-induced OA in mice^39^. Resveratrol inhibited the development of OA in an animal model via TLR4 and the PI3/Akt pathway^35^. We also showed that TLR4 was expressed in the joints of an MIA-induced model, which is consistent with a previous report^40^. Therefore, TLR4 could be a plausible target for the progression of arthritis induced by MIA. It may be possible to reduce the clinical symptoms of OA and structural joint damage of by inhibiting TLR4 signaling cascades.

TAP2 was initially reported as an antagonistic peptide of TLR4, having a strong anti-inflammatory effect on LPS-elicited RAW264.7 cells by suppressing the activation of NF-κB and MAPKs in TLR4-mediated signal transduction^12^. Interestingly, in our study, TAP2 was also effective in reducing the progression of MIA-induced arthritis in rats. Several processes could explain the attenuation of the cartilage damage by TAP2. First, chronic inflammatory processes involving TLR4 are mediated via a complex cytokine network. It is not yet entirely understood which intermediary factors are responsible for cartilage degradation and pain propagation. We found that TAP2 could reduce the production of proinflammatory cytokines, such as TNFα, IL-1β, and MMP13, from LPS-stimulated human chondrocytes. Thus, this peptide antagonist might work on cartilage by decreasing several important cytokines and MMP from chondrocytes and synoviocytes through TLR4 inhibition. Next, oxidative stress, including ROS, in the inflamed joints has also been implicated as a mediator of arthritis. High levels of ROS can lead to direct damage of the chondrocytes and cartilage matrix. MIA can induce ROS production in chondrocytes. TLR4 can stimulate ROS production via NADPH oxidases^41^, triggering proinflammatory cytokine production and subsequent OA pathogenesis^42^. In this study, TAP2 also reduced ROS in arthritic cartilage by MIA, which could be another mechanism of cartilage protection.

It is known that TLR4 signaling in astrocytes and microglia contributes to pain^43^. Previously, we proved that a single intrathecal injection of TAP2 decreased microglial activation^13^. We began TAP2 treatment on day 7 of the MIA treatment when signs of OA and cartilage degeneration were already present and TAP2 alleviated the pain. This suggests that TLR4 is involved in the pain sensitization of OA. Interestingly, TLR4 inhibition affected spinal astrocytes or microglia^13,44^. Spinal microglia contribute to the development of chronic pain, while astrocytes contribute to the maintenance of chronic pain^45,46^, possibly explaining how TLR4 inhibition can exert analgesic effects on both acute and chronic pain.

In addition to OA, TLR4 is implicated in other musculoskeletal diseases, such as inflammatory arthritis, disc degeneration, and peripheral neuropathy^11,47,48^. Therefore, TLR4 inhibition has been investigated for managing pain in models of arthritic pain^49^, disc degeneration^44^, and neuropathic pain^50^. Recently, a chemical TLR4 antagonist, TAK-232, showed good results in animal models of low back pain and disc degeneration^44^. Hence, we also compared TAP2 with other TLR4 inhibitors, such as C34 or M62812, and found that the duration of the analgesic effects was longer than those of these chemicals, which suggests that TAP2 is a potentially promising OA treatment. Therefore, TAP2 could be a good armament for managing pain in musculoskeletal diseases. Beyond cartilage protection, targeting the accompanying pain is likely more appropriate for the management of OA.

## Conclusion

We demonstrated that TAP2, a TLR4 inhibitor, decreased cartilage loss and pain in an MIA–induced arthritic rat model and proinflammatory cytokine and MMP13 in the animal model and human joint cells. Therefore, TAP2 can be a potential disease-modifying drug to slow cartilage degeneration and to provide pain relief for patients with OA.

## Supplementary information

Supplementary Information.

## References

[1] O'Neill TW, McCabe PS, McBeth J (2018). Update on the epidemiology, risk factors and disease outcomes of osteoarthritis. Best Pract. Res. Clin. Rheumatol..

[2] McAlindon TE (2014). OARSI guidelines for the non-surgical management of knee osteoarthritis. Osteoarthr. Cartil..

[3] Kloppenburg M (2019). 2018 update of the EULAR recommendations for the management of hand osteoarthritis. Ann. Rheum. Dis..

[4] Simon LS (2013). Nonsteroidal anti-inflammatory drugs and their risk: a story still in development. Arthr. Res. Ther..

[5] McAlindon TE (2017). Effect of intra-articular triamcinolone vs saline on knee cartilage volume and pain in patients with knee osteoarthritis: a randomized clinical trial. JAMA.

[6] Kumar H, Kawai T, Akira S (2009). Pathogen recognition in the innate immune response. Biochem. J..

[7] Xie DL, Meyers R, Homandberg GA (1992). Fibronectin fragments in osteoarthritic synovial fluid. J. Rheumatol..

[8] Campo GM (2010). Small hyaluronan oligosaccharides induce inflammation by engaging both toll-like-4 and CD44 receptors in human chondrocytes. Biochem. Pharmacol..

[9] Schelbergen RF (2012). Alarmins S100A8 and S100A9 elicit a catabolic effect in human osteoarthritic chondrocytes that is dependent on Toll-like receptor 4. Arthr. Rheumatol..

[10] Kim HA (2006). The catabolic pathway mediated by Toll-like receptors in human osteoarthritic chondrocytes. Arthr. Rheumatol..

[11] Ospelt C (2008). Overexpression of toll-like receptors 3 and 4 in synovial tissue from patients with early rheumatoid arthritis: toll-like receptor expression in early and longstanding arthritis. Arthr. Rheumatol..

[12] Park S (2017). TLR4/MD2 specific peptides stalled in vivo LPS-induced immune exacerbation. Biomaterials.

[13] Yin Y (2019). Analgesic effect of toll-like receptor 4 antagonistic peptide 2 on mechanical allodynia induced with spinal nerve ligation in rats. Exp. Neurobiol..

[14] Thakur M, Rahman W, Hobbs C, Dickenson AH, Bennett DL (2012). Characterisation of a peripheral neuropathic component of the rat monoiodoacetate model of osteoarthritis. PLoS ONE.

[15] Chung JM, Kim HK, Chung K (2004). Segmental spinal nerve ligation model of neuropathic pain. Methods Mol. Med..

[16] Zhang E (2013). Expression of LC3 and Beclin 1 in the spinal dorsal horn following spinal nerve ligation-induced neuropathic pain. Brain Res..

[17] Pitcher GM, Ritchie J, Henry JL (1999). Paw withdrawal threshold in the von Frey hair test is influenced by the surface on which the rat stands. J. Neurosci. Methods.

[18] Ito S, Suto T, Saito S, Obata H (2018). Repeated administration of duloxetine suppresses neuropathic pain by accumulating effects of noradrenaline in the spinal cord. Anesth. Analg..

[19] Gao YJ, Zhang L, Ji RR (2010). Spinal injection of TNF-alpha-activated astrocytes produces persistent pain symptom mechanical allodynia by releasing monocyte chemoattractant protein-1. Glia.

[20] Pritzker KP (2006). Osteoarthritis cartilage histopathology: grading and staging. Osteoarthr. Cartil..

[21] Cho IH (2008). Role of microglial IKKbeta in kainic acid-induced hippocampal neuronal cell death. Brain.

[22] Livak KJ, Schmittgen TD (2001). Analysis of relative gene expression data using real-time quantitative PCR and the 2(-Delta Delta C(T)) method. Methods.

[23] de Lange-Brokaar BJ (2016). Evolution of synovitis in osteoarthritic knees and its association with clinical features. Osteoarthr. Cartil..

[24] Kalaitzoglou E, Griffin TM, Humphrey MB (2017). Innate Immune Responses and Osteoarthritis. Curr. Rheumatol. Rep..

[25] Gomez R, Villalvilla A, Largo R, Gualillo O, Herrero-Beaumont G (2015). TLR4 signalling in osteoarthritis–finding targets for candidate DMOADs. Nat. Rev. Rheumatol..

[26] Bondeson J, Wainwright SD, Lauder S, Amos N, Hughes CE (2006). The role of synovial macrophages and macrophage-produced cytokines in driving aggrecanases, matrix metalloproteinases, and other destructive and inflammatory responses in osteoarthritis. Arthr. Res. Ther..

[27] Blom AB (2004). Synovial lining macrophages mediate osteophyte formation during experimental osteoarthritis. Osteoarthr. Cartil..

[28] Barreto G (2013). Do changing toll-like receptor profiles in different layers and grades of osteoarthritis cartilage reflect disease severity?. J. Rheumatol..

[29] Bobacz K (2007). Toll-like receptors and chondrocytes: the lipopolysaccharide-induced decrease in cartilage matrix synthesis is dependent on the presence of toll-like receptor 4 and antagonized by bone morphogenetic protein 7. Arthr. Rheumatol..

[30] Creamer P, Hunt M, Dieppe P (1996). Pain mechanisms in osteoarthritis of the knee: effect of intraarticular anesthetic. J. Rheumatol..

[31] Maqboul A, Elsadek B (2018). Expression profiles of TRPV1, TRPV4, TLR4 and ERK1/2 in the dorsal root ganglionic neurons of a cancer-induced neuropathy rat model. PeerJ.

[32] Miller RE (2015). Damage-associated molecular patterns generated in osteoarthritis directly excite murine nociceptive neurons through Toll-like receptor 4. Arthr. Rheumatol..

[33] Barochia A, Solomon S, Cui X, Natanson C, Eichacker PQ (2011). Eritoran tetrasodium (E5564) treatment for sepsis: review of preclinical and clinical studies. Expert Opin. Drug Metab. Toxicol..

[34] Neal MD (2013). Discovery and validation of a new class of small molecule Toll-like receptor 4 (TLR4) inhibitors. PLoS ONE.

[35] Xu X (2019). Resveratrol inhibits the development of obesity-related osteoarthritis via the TLR4 and PI3K/Akt signaling pathways. Connect Tissue Res..

[36] Chen YJ (2013). EGb761 inhibits inflammatory responses in human chondrocytes and shows chondroprotection in osteoarthritic rat knee. J. Orthop. Res..

[37] Nasi S (2014). Dispensable role of myeloid differentiation primary response gene 88 (MyD88) and MyD88-dependent toll-like receptors (TLRs) in a murine model of osteoarthritis. Jt. Bone Spine.

[38] Wang H (2018). Histomorphology and innate immunity during the progression of osteoarthritis: Does synovitis affect cartilage degradation?. J. Cell Physiol..

[39] Kalaitzoglou E (2019). TLR4 promotes and DAP12 limits obesity-induced osteoarthritis in aged female mice. JBMR Plus.

[40] Zhang Y, Zeng Y (2019). Curcumin reduces inflammation in knee osteoarthritis rats through blocking TLR4 /MyD88/NF-kappaB signal pathway. Drug Dev. Res..

[41] Canton J, Grinstein S (2014). Priming and activation of NADPH oxidases in plants and animals. Trends Immunol..

[42] Lepetsos P, Papavassiliou AG (2016). ROS/oxidative stress signaling in osteoarthritis. Biochim. Biophys. Acta.

[43] Nicotra L, Loram LC, Watkins LR, Hutchinson MR (2012). Toll-like receptors in chronic pain. Exp. Neurol..

[44] Krock E, Millecamps M, Currie JB, Stone LS, Haglund L (2018). Low back pain and disc degeneration are decreased following chronic toll-like receptor 4 inhibition in a mouse model. Osteoarthr. Cartil..

[45] Svensson CI, Brodin E (2010). Spinal astrocytes in pain processing: non-neuronal cells as therapeutic targets. Mol. Interv..

[46] Kawasaki Y (2008). Distinct roles of matrix metalloproteases in the early- and late-phase development of neuropathic pain. Nat. Med..

[47] Krock E (2017). Toll-like receptor activation induces degeneration of human intervertebral discs. Sci. Rep..

[48] Li Y (2015). The cancer chemotherapeutic paclitaxel increases human and rodent sensory neuron responses to TRPV1 by activation of TLR4. J. Neurosci..

[49] Christianson CA (2011). Spinal TLR4 mediates the transition to a persistent mechanical hypersensitivity after the resolution of inflammation in serum-transferred arthritis. Pain.

[50] Tanga FY, Nutile-McMenemy N, DeLeo JA (2005). The CNS role of Toll-like receptor 4 in innate neuroimmunity and painful neuropathy. Proc. Natl. Acad. Sci. USA.

