# Supplementary material for: TAP2, a peptide antagonist of Toll-like receptor 4, attenuates pain and cartilage degradation in a monoiodoacetate-induced arthritis rat model
Source: Sci Rep. 2020 Oct 15;10:17451. doi: 10.1038/s41598-020-74544-5 (PMC7567100; doi:10.1038/s41598-020-74544-5)
Supplement: Supplementary file 1 — Supplementary Information. [file 41598_2020_74544_MOESM1_ESM.pdf]

Supplementary information for

TAP2, a peptide antagonist of Toll-like receptor 4, attenuates pain and cartilage degradation in a monoiodoacetate-induced arthritis rat model

Hyewon Park[1,2], Jinpyo Hong[3], Yuhua Yin [1,2], Yongbum Joo[4], Youngmo Kim[4], Juhee Shin[1,2], Hyeok Hee Kwon[1,2], Nara Shin[1,2], Hyo Jung Shin[1,2], Jaewon Beom[5], Dong Woon Kim[1,2], Jinhyun Kim[6]

[1]Department of Medical Science, Chungnam National University College of Medicine, Daejeon, Republic of Korea.

[2]Department of Anatomy, Brain Research Institute, Chungnam National University College of Medicine, Daejeon, Republic of Korea.

[3]Department of Neuroscience and Physiology, and Dental Research Institute, Seoul National University School of Dentistry, Seoul, Republic of Korea.

[4]Department of Orthopedics, Chungnam National University College of Medicine, Daejeon, Republic of Korea.

[5]Department of Rehabilitation Medicine, Seoul National University Bundang Hospital, Seongnam, Gyeonggi-do, Republic of Korea.

[6]Department of Internal Medicine, Chungnam National University College of Medicine, Daejeon, Republic of Korea.

Supplementary Figure S1.

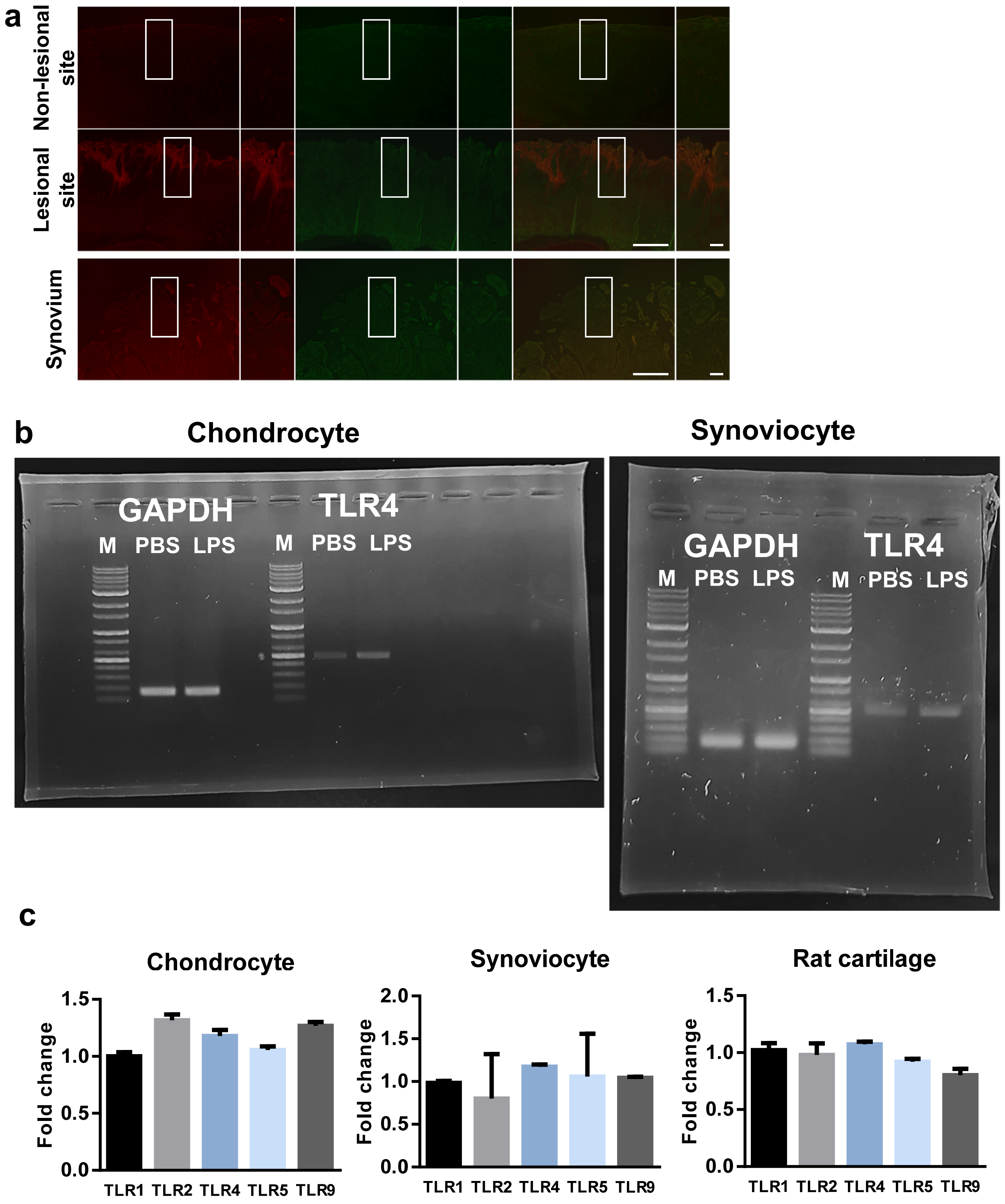

Figure S1 (a) Negative control staining of Fig. 1a. **(b)** Picture of whole gel of Fig. 1c. TLR4 inductions in LPS-stimulated primary human chondrocytes and synoviocytes were determined by RT-PCR. GAPDH was used as a control. **(c)** The expression of TLR1, TLR2, TLR3, TLR4 and TLR9 were analyzed by quantitative PCR in human chondrocyte and synoviocyte of OA joints and MIA-induced arthritic joint of rat.

Supplementary Figure S2.

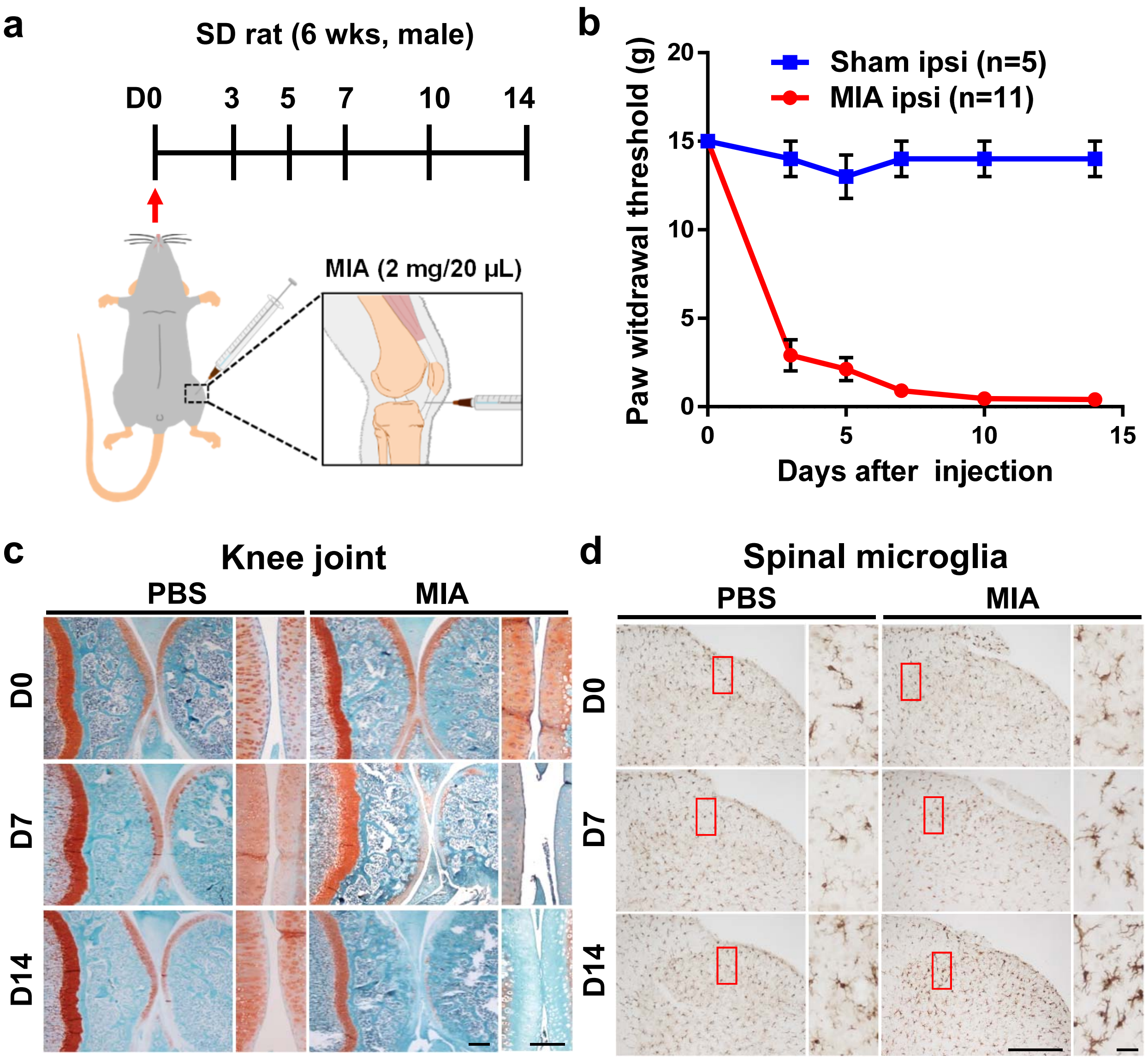

Figure S2 An osteoarthritic pain model was established by a single intraarticular injection of MIA to rats. **(a)** To prepare the OA pain model in rats, MIA was administered to the intraarticular space of male rats (6 weeks) by an injection of MIA (2 mg in 25 μl PBS). **(b)** The MIA-promoted pain was evaluated with von Frey filaments in the PBS- or MIA-treated groups. **(c)** Isolated knee joints at day 0, 7, and 14 post-MIA injection were decalcified and stained with Fast green and Safranin O to investigate the loss of knee cartilage caused by MIA. Scale bar = 1 mm (left), 100 μm (right). **(d)** Further, the activity and proliferation of microglia in the ipsilateral sides of the spinal dorsal horns at days 0, 7, and 14 following PBS or MIA delivery were also investigated by immunostaining with anti-Iba1 antibodies. Scale bar = 100 μm (left), 50 μm (right)..

Supplementary Figure S3.

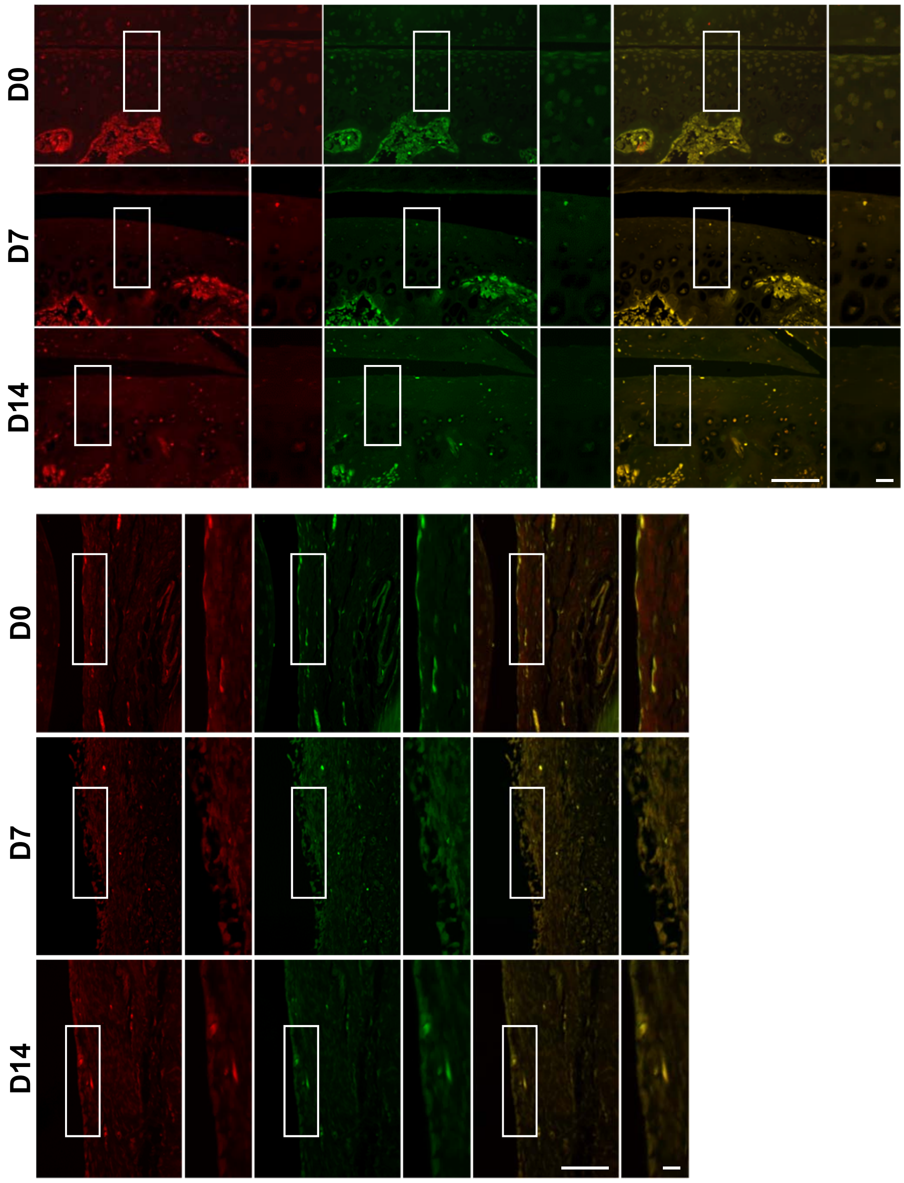

Figure S3 Negative control staining of Figs. 2a, b.

Supplementary Figure S4.

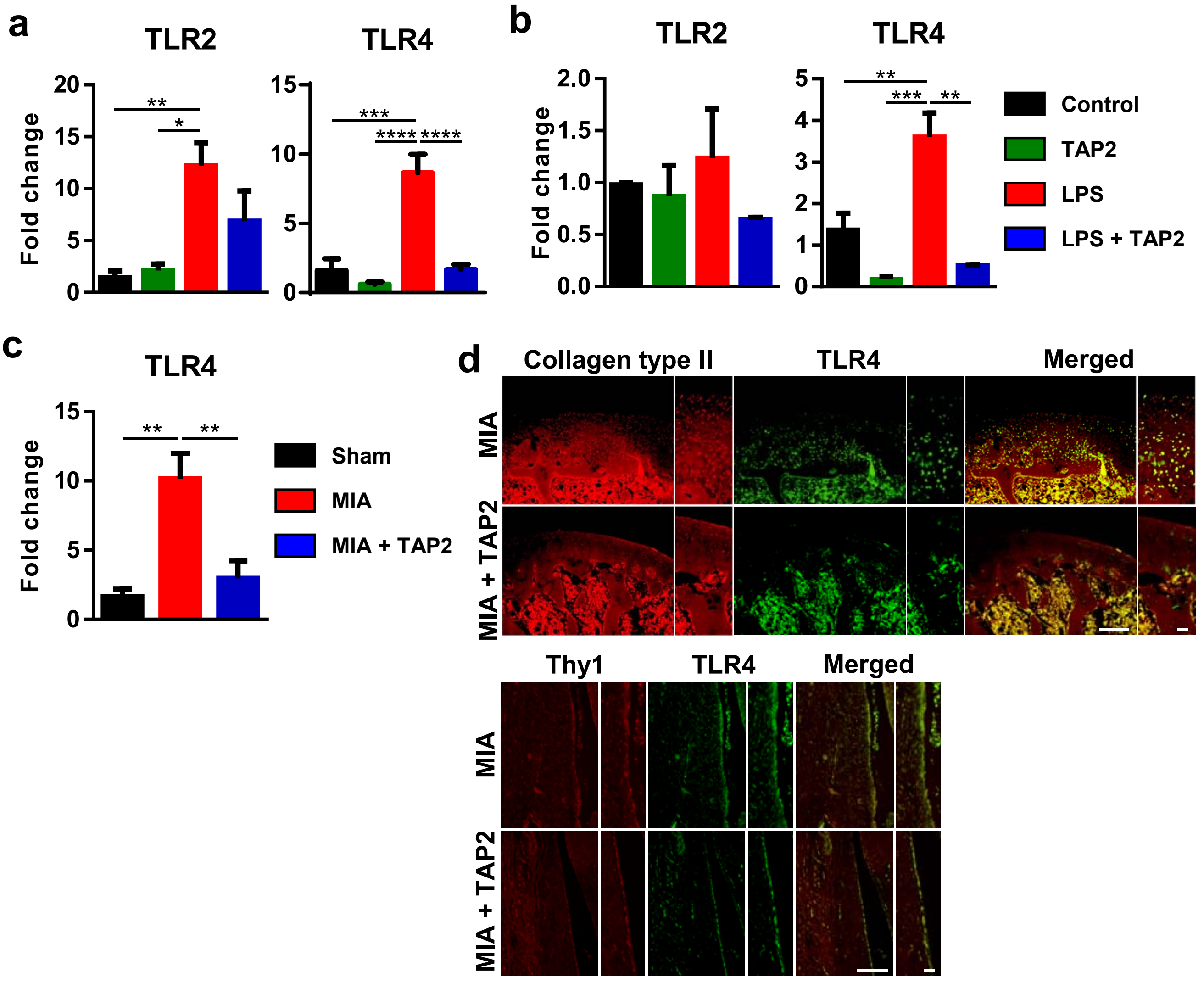

Figure S4 The changes of TLR2 and TLR4 expression by TAP2 treatment in chondrocyte and synoviocyte. **(a)** Primary human chondrocytes of OA joints were incubated with TAP2 (10 μM) or PBS for 3 hours prior to LPS treatment (0.1 ng/ml, 3 h) or **(b)** human synoviocytes of OA joints was incubated with TAP2 (100 μM) or PBS for 3 hours prior to LPS treatment (100 ng/ml, 3 h). Then, total RNA was isolated from the cells and utilized for the analysis of TLR2 or TLR4 by quantitative PCR. The data are expressed as the mean ± SEM (one-way ANOVA test, $^{****}P < 0.0001$, $^{**}P < 0.01$ vs. LPS + TAP2). **(c)** On day 3 post-injection of PBS or TAP2, total RNA from the knee joint cartilage of MIA-induced arthritic rats, MIA arthritis rats treated with TAP2, and control animals was isolated and analyzed by quantitative PCR (n=3 per each group, one-way ANOVA test, $^{**}P < 0.01$ vs. MIA + TAP2). **(d)** MIA-induced rats were injected with PBS or TAP2 into the knee joint on day 7. The tissues of knee joints were co-immunostained with anti-TLR4 antibodies and anti-collagen type II antibodies (chondrocyte marker) or anti-TLR4 antibodies and anti-Thy1 antibodies (synoviocyte marker). Scale bar = 100 μm.
